# Which Devour Widows' Houses

**Published:** 1919-10-18

**Authors:** 


### WHICH DEVOUR WIDOWS' HOUSES.

BY A SOCIAL WORKER.

The devourer in this case has the best possible intentions. It is, in fact, that expression of the national conscience which recognises a duty to the widow and the orphan and is embodied in the pious or official builders of great institutions for the children. These are handsomely designed, lavishly equipped, liberally staffed; in comparison with the children's homes they are palaces, and the boys and girls receive most competent physical love and a sound education. They want for nothing; indeed, it was said some years ago of the inmates of a certain Poor-Law institution that village children envied their sweet-buying powers. Not the State alone, but private charity often the result of real self-denial, has provided these refuges for the orphan, and the standard of life in them has grown ever higher and higher. Yet experience has shown that something is wanting, an elusive something which our own generation recognises as being just the difference between a *Home* and a *home*. The trend of thought was shown when the old type of barrack school, such as George Müller's orphanages at Bristol, was replaced by the Barnardo village, where thirty inmates of the cottages were reduced to fifteen or twelve, and children attended the ordinary elementary school. Then boarding-out in private families was tried, and vigorously pressed for by the State Children's Association and other workers of experience. Now we are told that the Independent Order of Oddfellows have determined upon a scheme by which the orphans of their members will all be placed in suitable family circles. It has taken many years to learn the rather obvious lesson that childhood is not an isolated thing, but the first volume of a life-story, and that according to the early development of character will be the later action, when the plot thickens and the influence of other lives and stress of circumstances have to be faced.

### Good Homes and Good Mothers.

These are, of course, now truisms of psychology, but it is extraordinary that in a day when so much is constantly said about the value to the nation of good homes and good mothers the suffering and injury inflicted upon a number of homes and of mothers by removing the children or giving a scanty out-relief, which in either case compels the woman to work are disregarded. Recently, this has been recognised by the pensions given to widows and orphans of men who have fallen in the late war. But this plan takes no account of all those who are in the same case, except that the lost bread-winner was a civilian, or a soldier who died from the hardships of the Boer War or was killed in some frontier "scrap" hardly noticed in the newspapers, or, again, was overstrained by serving his country, not in the ranks, but in some skilled craft which he was not allowed to leave for Army service. And yet, in addition to all other reasons, it is actually less expensive—as has been found in America—to entrust the children to their own mother's care, as the wise Princess of Egypt entrusted her foundling from the Nile. Estimates of cost are, of course, at present very difficult to make, but the Executive of the State Children's Association say truly that since in some of the Metropolitan Poor-Law schools a child is now costing 20s. a week, if the mother were given the present allowance for soldiers' families for herself and three children, the State would pay 36s. 6d., a money saving of 23s. 6d. weekly. It is no doubt unfortunate that the idea of instituting in this country such pensions for penniless widows and their children under school age is often confused with the very different idea of general endowment of motherhood. Different, indeed, for while the one system, adopted now in thirty-five out of the forty-eight United States of America, aims at maintaining the home with its personal responsibilities, the other regards child-bearing as service to the country, to be paid for accordingly, and, as Dr. Christine Murrell lately owned, while vigorously advocating it, at a huge cost.

### The Delinquent Child.

Meantime the question of the delinquent child is attracting more and more attention. It is comforting to be told that in 1918 the considerable number of 49,896 cases brought before juvenile courts showed a decrease of 2,404 on the figures of 1917. This decrease is certainly largely due to preventive efforts, and it is very interesting to hear some first suggestions of the advisability of doing away with the paraphernalia of courts and committing young offenders to the care of the Board of Education. Many grown-ups forget, or have never known, what the excitement and romance of notable naughtiness mean to some children as a change from the routine of daily life. The experiment tried by Mr. Clarke Hall, Magistrate of Old Street Children's Court in London, showed lately how much could be done by personal interest and care. Sub-committees were appointed by the Juvenile Organisations Committees of the eight Boroughs forming his district and comprising about a million persons, and these chose voluntary workers, who each took charge of one or two children on probation, being themselves responsible to the court's two salaried Probation Officers. Now, among delinquent children many are always found to be children of women who go out to work, so that they lack home care and training. The publication by the State Children's Association of an article on Judge Neil's campaign for widows' pensions brought a number of letters from a class hitherto suffering silently. "It seems a crime to be a respectable widow," wrote one who worked from six to four, having parted with three children, to keep herself and the other three, all being under ten. Another, with eight boys and two girls, had had five serious operations, and yet was struggling to keep her four youngest. (Good people constantly reproach the working woman for beginning to dread a large family.)

The State Children's Association has now drafted a scheme on which a Bill might be founded, and asks for help in promoting legislation. Widows without income, apart from earnings which does not exceed the amount of pension on the scale of separation allowance to soldiers' wives and having one or more dependent children under school-leaving age, should have a right to this pension. No new authority should be set up or new visitors appointed; existing machinery is ample and suitable. Should the mother prove an unfit guardian, foster-parents should be appointed. For the present it is proposed to confine the scheme entirely to widows, but three classes of women might ultimately be included. These are: Deserted wives, wives of permanently incapacitated men, and those of long-sentence prisoners. These last would seem specially to deserve consideration, since the children may well need careful watching and training.

Can any one raise any valid objections to so sound and simple a policy?

